# Overestimation of clinical N-staging in microsatellite instable gastric cancers is associated with VEGF-C signaling and CD8+ T-cell dynamics

**DOI:** 10.1093/oncolo/oyae288

**Published:** 2024-11-18

**Authors:** Chun-Yi Tsai, Tzong-Shyuan Tai, Shih-Chiang Huang, Tsung-Hsing Chen, Jun-Te Hsu, Chun-Nan Yeh, Ying-Chieh Lai, Gigin Lin, Ta-Sen Yeh

**Affiliations:** Department of Surgery, Chang Gung Memorial Hospital at Linkou, Chang Gung University College of Medicine, Taoyuan 333, Taiwan; Department of Medical Research and Development, Chang Gung Memorial Hospital at Linkou, Chang Gung University College of Medicine, Taoyuan 333, Taiwan; Department of Pathology, Chang Gung Memorial Hospital at Linkou, Chang Gung University College of Medicine, Taoyuan 333, Taiwan; Department of Gastroenterology, Chang Gung Memorial Hospital at Linkou, Chang Gung University College of Medicine, Taoyuan 333, Taiwan; Department of Surgery, Chang Gung Memorial Hospital at Linkou, Chang Gung University College of Medicine, Taoyuan 333, Taiwan; Department of Surgery, Chang Gung Memorial Hospital at Linkou, Chang Gung University College of Medicine, Taoyuan 333, Taiwan; Department of Radiology, Chang Gung Memorial Hospital at Linkou, Chang Gung University College of Medicine, Taoyuan 333, Taiwan; Department of Radiology, Chang Gung Memorial Hospital at Linkou, Chang Gung University College of Medicine, Taoyuan 333, Taiwan; Department of Surgery, Chang Gung Memorial Hospital at Linkou, Chang Gung University College of Medicine, Taoyuan 333, Taiwan

**Keywords:** microsatellite instable gastric cancer, clinical N-staging, lymph node ratio, VEGF-C, CD8+ T cell

## Abstract

**Background:**

Microsatellite instable (MSI) gastric cancers exhibit reduced lymph node (LN) metastasis and improved survival compared to microsatellite stable (MSS) counterparts. However, to our longstanding observation, clinical N-staging (cN) is frequently overestimated in MSI cases. The clinical implications and underlying mechanisms of this discrepancy warrant further investigation.

**Materials and methods:**

We conducted a comprehensive review of clinicopathological data from a 141 MSI and 1119 MSS gastric cancer patients. Expression of vascular endothelial growth factor-C (VEGF-C) and its receptor VEGFR-3 were assessed using qPCR and immunohistochemistry. High-parameter flow cytometry was employed to analyze subsets of CD8+ T cells within the tumors.

**Results:**

Multivariate analysis revealed that MSI status was an independent prognostic factor, alongside the LN ratio and AJCC8 pathology staging. MSI gastric cancers exhibited a reduced LN ratio, particularly at advanced T-staging, compared to MSS counterparts, while maintaining an equivalent LN yield. Overestimation of cN by computed tomography preoperatively was frequent in MSI gastric cancers but was more commonly underestimated in MSS counterparts. VEGF-C and VEGFR-3 expression were lower in MSI tumors. MSI gastric cancers showed an increased total number of CD8+ T cells, albeit with a lower proportion of effector memory cells expressing CD45RA (EMRA) and CD8+ CXCR4+ T cells, compared to MSS counterparts.

**Conclusion:**

Frequent overestimation of clinical N-staging in MSI gastric cancers is associated with VEGF-C signaling and CD8+ T-cell dynamics and should be cautiously interpreted, as it might misguide therapeutic options.

## Teaser

This study examined the phenomenon and mechanisms of clinical N-staging over-estimation in microsatellite instable gastric cancers, particularly at advanced T-staging. By investigating VEGF-C signaling and CD8+ T-cell dynamics within the tumor microenvironment.

**Implications for practice**

Caution should be exercised regarding the overestimation of cN in MSI gastric cancers, particularly at advanced T-staging, as it might misguide therapeutic algorithms. Through the modulation of VEGF-C signaling and CD8+ T-cell dynamics, MSI gastric cancer patients appear to resist rapid lymphatic metastasis and immune evasion.

## Introduction

Gastric cancer is a significant global health concern, ranking as the fifth most prevalent cancer and the second leading cause of cancer-related deaths worldwide. In 2014, the Cancer Genome Atlas (TCGA) and the Asian Cancer Research Group (ACRG) introduced genomic classifications for gastric cancers, highlighting their notorious heterogeneity.^1,2^ Within these classifications, microsatellite instability (MSI) emerged as a distinct molecular subgroup characterized by a hyper-mutable phenotype due to frequent polymorphisms in short, repetitive DNA sequences and single nucleotide substitutions, attributed to defective DNA mismatch repair machinery.^3^ A growing body of evidence has revealed that MSI gastric cancer is associated with better survival due to fewer nodal metastases compared to the MSS counterpart^4^; however, the mechanism remains enigmatic. Caution is warranted regarding the use of fluorouracil-based adjuvant chemotherapy for MSI gastric cancers.^5^ Instead, MSI gastric cancer presents itself as an appealing candidate for immunotherapy using immune checkpoint inhibitors.^6,7^

While multidetector computed tomography (MD CT) remains essential for clinical staging, its accuracy in predicting clinical N-category (cN) has been questioned. Our longstanding observation indicates that cN of MSI gastric cancers tends to be overestimated by MD CT, which could significantly misguide subsequent management strategies. The gold standard for gastric cancer pathology staging, management algorithm, and prognosis prediction following resection is the tumor-node-metastasis (TNM) stage proposed by UICC/AJCC. However, the variability in clinical outcomes among patients with the same TNM stage has prompted the development of ancillary classifications such as the WHO and Lauren’s classifications, as well as various genetic profiles for more precise outcome prediction and management modifications. For instance, the presence of tumor-infiltrating lymphocytes (TILs) within the tumor microenvironment (TME) has been advocated for its better prognostic value beyond TNM classification.^8^

In the last decade, cancer immunotherapy, particularly immune checkpoint blockade, has revolutionized cancer management, including gastric cancer, where CD8+ cytotoxic T cells play a central role in this approach. Meanwhile, tumor cells employ various immunosuppressive factors, including immune checkpoint molecules like CTLA4 and PD-1, as well as immunosuppressive cells like regulatory T cells, myeloid-derived suppressor cells, and tumor-associated macrophages (TAMs).^9^ TAMs not only produce immunosuppressive cytokines such as TGFβ and IL-10 but also VEGFs (vascular endothelial growth factors). Of them, VEGF-C enhances lymphangiogenesis and lymph node metastasis by binding to the receptor VEGFR-3 in an autocrine and paracrine manner.^10-13^ Apart from transporting tumor cells to lymph nodes, lymphatic endothelial cells lining lymphatic vessels actively participate in adaptive immunity, highlighting the dual role of VEGF-C signaling in promoting tumorigenesis both physically and immunologically.^14-16^

In the present study, our aim was to elucidate the phenomenon and mechanisms of cN over-estimation in MSI gastric cancers, particularly at advanced T-staging. By investigating VEGF-C signaling and CD8+ T-cell dynamics within the TME, we seek to understand how MSI gastric cancers leverage these pathways to escape from rapid disease progression and immune evasion.

## Patients and methods

This study was approved by the institutional review board of Chang Gung Memorial Hospital at Linko (CGMH). Patients who had undergone preoperative (neoadjuvant) therapy were excluded from this study. A total of 141 patients with MSI gastric cancer and 1119 patients with MSS gastric cancer, who had undergone curative-intent gastrectomy from 1999 to 2020 were enrolled. Data including demographics, clinical and pathological stages, pathological parameters, and microsatellite status were obtained from medical records. cN derived from MD CT of the patients studied were independently reviewed by two radiologists (YCL and GGL) who adhered to the diagnostic criteria of nodal metastasis described elsewhere.^17^ Pathological N-stage (pN) was designated according to the AJCC staging Manual, edition 8. Lymph node (LN) yield was defined as number of lymph node retrievals during gastrectomy. LN ratio was defined as the ratio of metastatic to retrieved lymph nodes.

The standard adjuvant chemotherapy regimens were as follow: stage I and II patients received TS1 orally, while stage III patients were treated with Xelox plus cisplatin (or oxaliplatin). Up to one-third of the patients did not receive adjuvant chemotherapy due to patient preference, advanced age, and/or frailty. Disease free survival (DFS) was evaluated from the date of surgery to the date of first relapse identified. The last outcome review was set on December 31, 2021.

## Microsatellite status detected by immunohistochemistry

The formalin-fixed and paraffin-embedded tissue samples stored in archives of Department of Pathology of CGMH were used for DNA MMR (mismatch repair) protein assay including MLH1 (1:50, Genemed), MSH2 (1:100, Zeta, Arcadia), MSH6, (1:150, Abcam), and PMS2 (1:100, BD Pharmingen). Those with and without absence of any MMR protein (s) were defined as MSI and MSS, respectively.

## Quantitative real-time polymerase chain reaction (qPCR) for VEGF-C and VEGFR-3

Total RNA was extracted by using RNeasy RNA Mini Kit (Qiagen). The quantity of RNA samples was determined using NanoDrop One (Thermo Scientific). Total RNA samples were reverse- transcribed (Applied Biosystems). cDNA derived from 10 ng RNA was used for Quantitative PCR performed using 2X TaqMan Gene Expression Master Mix (Applied Biosystems) and TaqMan probe assay. Each assay was run on an Applied Biosystems 7900HT Real-Time PCR system in triplicates and expression fold-changes were derived using the comparative CT method, GAPDH as endogenous control.

## IHC for VEGF-C, VEGFR-3, CD8+, and CXCR4

FFPE (formalin-fixed paraffin-embedded) tissues were cut into 4-μm sections and mounted on the glue-coated slides. A modified avidin-biotin-peroxidase complex method was performed. Various primary antibodies against VEGF-C (1:100, Santa Cruz), VEGFR-3 (1:100, Abcam), CD8+ (1:100, Abcam), and CXCR4 (1:100, Abcam) were applied. Cytoplasmic staining intensity of VEGF-3, VEGFR-3, and CXCR4 was graded from 0 to 3, while the percentage of positive- stained cells (area proportion) was graded 0%, <25%, <50%, and >50%. A semi-quantitative analysis by taking intensity and area proportion into account was acquired and designated as weak, moderate, and strong signals. Nuclear stains of CD8+ was quantitatively analyzed by choosing 5 randomized visual fields per section at a magnification of ×200. The detailed procedures of the double staining technique toward CD8+ and CXCR4 was described elsewhere.^18^

## High-parameter flow cytometer

To explore potential phenotypic variations in lymphocytes between MSI and MSS, we employed a high-parameter flow cytometer to analyze the expression of 35 immune markers in the collected samples (Figure S1). Frozen-stored tumor samples retrieved during the surgeries were homogenized using the GentleMacs Dissociator following the manufacturer’s protocols. Single-cell suspension was subjected to Ficoll gradient, and the mononuclear cells were collected and frozen in liquid nitrogen for preservation. For high-parameter staining, the frozen cells were thawed by adding 10 mL of medium (RPMI1640 with 10% FBS) to recover them from the liquid nitrogen. Next, 5 × 10^5^ peripheral blood mononuclear cells were re-suspended in FASC staining buffer (PBS with 1% FBS) and stained with fluorochrome-conjugated antibodies on ice for 20 minutes. Flow cytometric analysis was performed using a FACSymphony A5.2 instrument (BD Bioscience) to acquire the data. The collected data were subsequently analyzed using FlowJo software (BD Bioscience) and the CATALYST package in R.

## Statistics

All continuous variables were expressed as mean ± standard deviation. Statistical analysis was performed with the Student’s *t* test for continuous data. Analysis of LN ratio and mRNA of VEGF-C and VEGFR-3 were sketched using a box plot, and data were expressed as medians. To analyze the LN ratio as one of the prognostic factors, we used recursive partitioning analysis, which creates a survival analysis tree and establishes optimal cutoff points that discriminate survival outcomes. DFS was estimated using the Kaplan-Meier methods and differences in survival distribution was assessed using the log rank test. Factors with *P* < .05 in the univariate analysis were then included in the multivariate analysis. The final multivariate model was determined using Cox proportional hazard regression. *P* < .05 was considered statistically significant. Statistical analyses were performed using SPSS for Windows, version 13 (SPSS, Inc.,).

## Results

### Clinicopathological and outcome analyses

**Figure 1.**
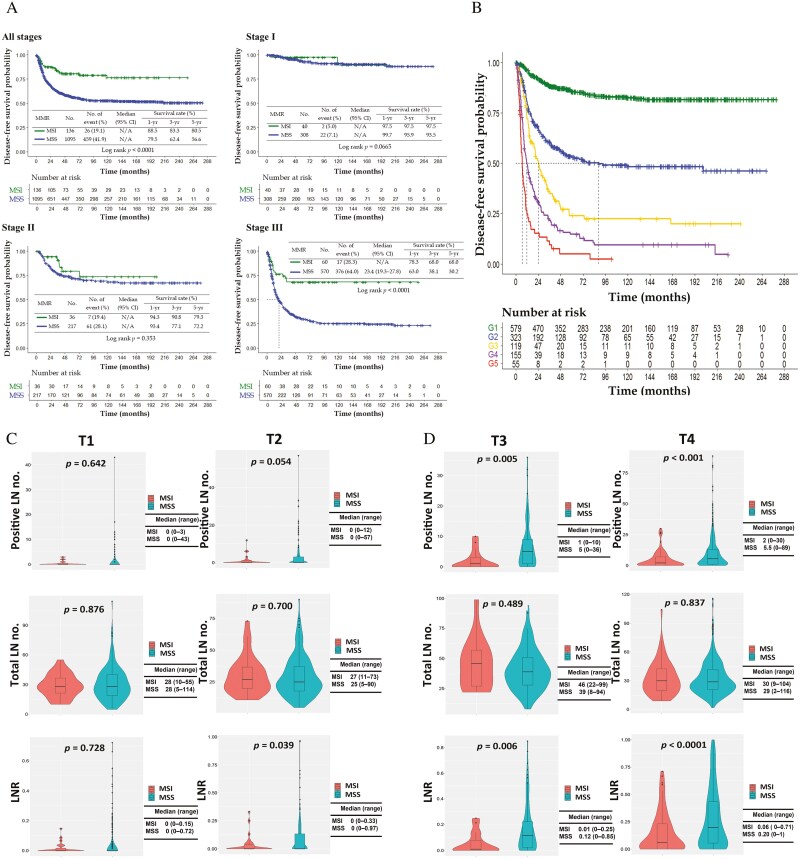
A. Disease-free survival of gastric cancer patients with MSI vs MSS stratified by pathological staging. B. Disease-free survival of gastric cancer patients stratified by lymph node ratio. G1 to G5, indicated ranges of lymph node ratio as <0.056, 0.056 to 0.257, 0.257 to 0.400, 0.400 to 0.739, and >0.739, respectively. *P* < 0.01, log-rank test. C. Metastatic lymph nodes, lymph node retrieval and lymph node ratio of T1- and T2-staging gastric cancers with MSI vs MSS. D. Metastatic lymph nodes, lymph node retrieval and lymph node ratio of T3- and T4-staging gastric cancers with MSI vs MSS. Abbreviations: MSI, microsatellite instable; MSS, microsatellite stable.

The MSI cohort was characterized by older age, tumor site at the distal stomach, and thus less execution of total gastrectomy, earlier N-stage, less LN ratio, earlier pathological stage, and less peri-neural invasion, as compared to MSS cohort (Table 1). Multivariate analysis showed that independent prognostic factors for DFS included LN ratio (hazard ratio [HR], 7.7 for the highest tier), pathological stage (HR, 2.5 for the highest tier), microsatellite status (HR, 1.8), tumor size (HR, 1.5), and total gastrectomy (HR, 1.3), in decreasing order (Table 2). DFS of patients with stage I-III MSI gastric cancers was superior to that of MSS counterpart (*P* < .0001), while sub-analysis showed this trend remained valid only in stage III gastric cancers (*P* < .0001) (Figure 1A). LN ratio represented the most robust prognosticator to predict DFS (Figure 1B). Next, we analyzed difference of LN ratio between MSI and MSS cohorts by each T-stage (Figures 1C and D). Despite an equivalent LN yield in both cohorts, the difference of LN ratio between MSI and MSS cohorts increased progressively with higher T-categories. The median value of LN ratio of MSI cohort at pT4 was 0.06, which was very close to the lowest of 5-tier classification; while the median value of LN ratio of MSS cohort at pT4 was 0.20, located at the second tier of 5-tier classification.

**Table 1.** Demographics and clinicopathological parameters among patients with microsatellite instable and microsatellite stable gastric cancers.

Parameters	Microsatellite status		*P*-value
	MSI	MSS	
No. of cases (%)	141 (11.2)	1119 (88.8)	
Age, median (range), years	73 (33–94)	65 (24–94)	<.0001
Sex, *n* (%)			.588
Male	84 (59.6)	693 (61.9)	
Female	57 (40.4)	426 (38.1)	
Type of resection, *n* (%)			<.0001
Total gastrectomy	14 (9.9)	314 (28.1)	
Partial gastrectomy	127 (90.1)	805 (71.9)	
Location, *n* (%)			<.0001
Upper	6 (4.3)	220 (19.7)	
Middle	16 (11.3)	198 (17.7)	
Lower	115 (81.6)	666 (59.5)	
Whole	3 (2.1)	30 (2.7)	
Others	1 (0.7)	5 (0.4)	
Tumor size (cm), median (range)	5.4 (1–27)	3.7 (0.2–26)	<.0001
Histology, *n* (%)			.204
Well	13 (9.2)	94 (8.4)	
Moderate	54 (38.3)	347 (31.0)	
Poor	53 (37.6)	408 (36.5)	
Signet-ring cell	19 (13.5)	241 (21.5)	
Others	2 (1.4)	29 (2.6)	
T stage, *n* (%)			.052
T1	23 (16.3)	246 (22.0)	
T2	30 (21.3)	168 (15.0)	
T3	17 (12.0)	91 (8.1)	
T4	71 (50.4)	614 (54.9)	
N stage, *n* (%)			.001
N0	69 (48.9)	417 (37.3)	
N1	28 (19.9)	158 (14.1)	
N2	21 (14.9)	196 (17.5)	
N3a	18 (12.8)	206 (18.4)	
N3b	5 (3.5)	142 (12.7)	
AJCC 8 staging, *n* (%)			<.0001
IA	17 (12.1)	195 (17.4)	
IB	25 (17.7)	117 (10.5)	
IIA	15 (10.6)	63 (5.6)	
IIB	24 (17.0)	159 (14.2)	
IIIA	38 (27.0)	244 (21.8)	
IIIB	16 (11.3)	198 (17.7)	
IIIC	6 (4.3)	143 (12.8)	
No. of lymph node retrieval, median (range)	29 (9–104)	29 (2–116)	.560
LNR, median (range)	0.02 (0–0.71)	0.09 (0–1)	<.0001
Lymphovascular invasion, *n* (%)	68 (48.2)	597 (53.4)	.251
Perineural invasion, *n* (%)	45 (31.9)	544 (48.6)	<.001

MSI and MSS represented microsatellite instable and microsatellite stable, respectively, based on status of mismatch repair (MMR) proteins (see Materials and methods). LNR, metastatic to retrieved lymph node ratio.

**Table 2.** Univariate and multivariate disease-free survival analysis of patients with gastric cancers.

Parameters	Univariate analysis			Multivariate analysis		
	Hazard ratios	95% CI	*P* value	Hazard ratios	95% CI	*P* value
Age				—		
≤65 (*n* = 605)	1					
>65 (*n* = 626)	1.02	0.85–1.22	.829			
Gender				—		
Male (*n* = 757)	1.10	0.92–1.33	.295			
Female (*n* = 474)	1					
Type of resection						
Total gastrectomy (*n* = 317)	1.79	1.48–2.17	<.0001	1.36	1.03–1.80	.028
Partial gastrectomy (*n* = 914)	1			1		
Location						
Upper (*n* = 220)	1.50	1.10–2.05	.011	0.92	0.66–1.30	.648
Middle (*n* = 208)	1			1		
Lower (*n* = 764)	1.21	0.93–1.57	.163	1.21	0.91–1.61	.189
Whole (*n* = 33)	3.34	2.08–5.36	<.0001	1.50	0.92–2.44	.103
Others (*n* = 6)	1.32	0.32–5.38	.700	1.06	0.26–4.34	.939
Tumor size (cm)						
≦2.8 (*n* = 385)	1			1		
>2.8 (*n* = 846)	3.98	3.07–5.16	<.0001	1.50	1.12–2.00	.006
Histology						
Differentiated (*n* = 495)	1			1		
Undifferentiated (*n* = 736)	1.51	1.25–1.82	<.0001	1.10	0.91–1.34	.321
T stage						
T1 (*n* = 264)	1			1		
T2 (*n* = 191)	2.24	1.33–3.79	.002	1.14	0.63–2.09	.660
T3 (*n* = 107)	4.29	2.48–7.43	<.0001	1.02	0.47–2.19	.963
T4 (*n* = 669)	10.08	6.62–15.36	<.0001	1.89	0.94–3.81	.075
N stage				-		
N0 (*n* = 479)	1					
N1 (*n* = 177)	3.30	2.31–4.72	<.0001			
N2 (*n* = 211)	5.62	4.10–7.72	<0.0001			
N3a (*n* = 219)	9.35	6.91–12.66	<.0001			
N3b (*n* = 145)	16.14	11.75–22.18	<.0001			
LNR						
≦0.056 (*n* = 579)	1			1		
>0.056, ≦0.257 (*n* = 323)	4.02	3.07–5.26	<.0001	2.00	1.32–3.02	.001
>0.257, ≦0.400 (*n* = 119)	7.73	5.66–10.56	<.0001	3.23	2.03–5.14	<.0001
>0.400, ≦0.739 (*n* = 155)	13.06	9.87–17.27	<.0001	5.02	3.20–7.86	<.0001
>0.739 (*n* = 55)	23.45	16.44–33.44	<.0001	7.75	4.70–2.77	<.0001
AJCC 8 stage						
I (*n* = 348)	1			1		
II (*n* = 253)	4.23	2.66–6.74	<.0001	2.10	1.09–4.04	.027
III (*n* = 630)	15.53	10.27–23.47	<.0001	2.54	1.05–6.13	.039
Microsatellite status						
MSI (*n* = 136)	1			1		
MSS (*n* = 1095)	2.52	1.70–3.74	<.0001	1.86	1.24–2.80	.003
Lymphovascular invasion						
No (*n* = 583)	1			1		
Yes (*n* = 648)	4.13	3.36–5.08	<.0001	0.99	0.76–1.30	.972
Perineural invasion						
No (*n* = 656)	1			1		
Yes (*n* = 575)	3.21	2.65–3.88	<.0001	1.03	0.83–1.29	.771

Abbreviations: CI, confidence interval; LNR, metastatic to retrieved lymph node ratio; MSI, microsatellite instable; MSS, microsatellite stable.

### Discordance between clinical and pathological N-staging

Discrepancies between cN detected by MD CT and pN among MSI and MSS gastric cancers were shown (Table 3). cN of MSS cohort was frequently underestimated over half of the cases; oppositely, cN of MSI was frequently overestimated, particularly for those with pN0- and pN1-staging by up to 53% and 46%, respectively. The prevalence of pT3N0 and pT4N0, respectively, in MSI cohort were much higher than MSS cohort (44% vs 17%, *P* = .015; and 28% vs 15%, *P* = 0.01; Table 4).

**Table 3.** Clinical^*^ versus pathological N-staging concordance of gastric cancers.

pN	MSI (n = 141)		MSS (n = 1119)		*P*-value
	Under-estimated	Over-estimated	Under-estimated	Over-estimated	
pN_0_	—	37 (53.6)	—	83 (19.9)	<.0001
pN_1_	3 (10.7)	13 (46.4)	91 (57.6)	26 (16.5)	<.0001
pN_2_	5 (23.8)	7 (33.3)	103 (52.6)	23 (11.7)	<.001
pN_3_	3 (13.0)	—	208 (59.8)	—	<.001

^*^Assessed by multi-detector computed tomography preoperatively.

pN, pathological N-staging based on AJCC 8.

Under- or over-estimation of clinical N-staging of gastric cancers was referenced to pN-staging.

In parenthesis were percentages.

Abbreviations: MSI and MSS represented microsatellite instable and microsatellite stable, respectively.

**Table 4.** Status of lymph node metastasis of gastric cancer stratified by T-stage.

T/N stage	Microsatellite status		*P*-value
	MSI	MSS	
No. of cases	141	1119	
T1			.594
N0	17 (73.9)	195 (79.3)	
N+	6 (26.1)	51 (20.7)	
T2			.095
N0	21 (70.0)	90 (53.6)	
N+	9 (30.0)	78 (46.4)	
T3			.032
N0	8 (47.1)	19 (20.9)	
N+	9 (52.9)	72 (79.1)	
T4			.005
N0	23 (32.4)	113 (18.4)	
N+	48 (67.6)	501 (81.6)	

N0 and N+ indicated negative and positive lymph node metastasis no matter how many, respectively.

In parenthesis are percentages.

Abbreviations: MSI, microsatellite instable; MSS, microsatellite stable.

### VEGF-C and VEGFR-3

**Figure 2.**
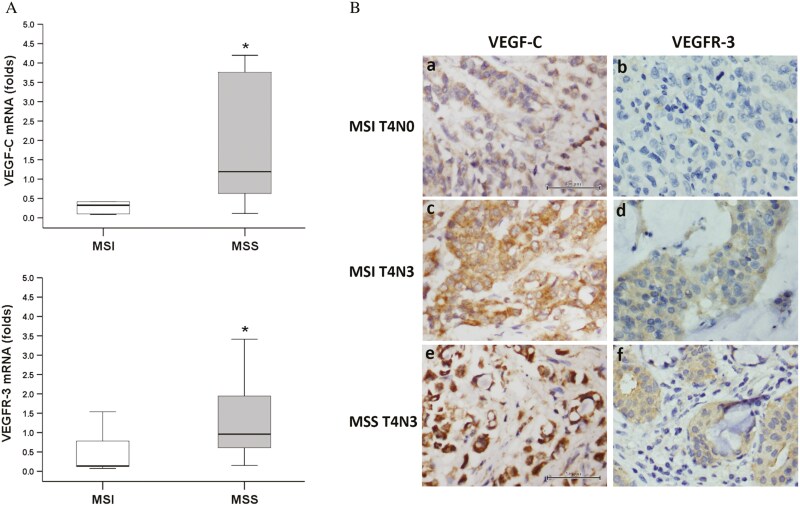
A. Expression of mRNA of VEGF and VEGFR-3 detected by qPCR among stage III gastric cancers with MSI (*n* = 11) vs MSS (*n* = 25). *Indicated *P* < .01. B. Representative expression of VEGF (a, c, e) and VEGF-R3 (b, d, f) detected by immunohistochemistry among gastric cancers with MSI T4N0, MSI T4N3, and MSS T4N3, respectively (n = 10 in each group). Abbreviations: VEGF, vascular endothelial growth factor; VEGFR-3, vascular endothelial growth factor- receptor 3.

The mRNA of VEGF-C and VEGFR-3 mRNAs of MSI stage III (pT4N+) gastric cancers detected by qPCR were both lower than MSS counterpart (Figure 2A). IHC revealed that VEGF-C was strongly expressed in MSS T4N3 gastric cancers, followed by MSI T4N3 gastric cancers and MSI T4N0 gastric cancers, in decreasing order. Meanwhile, VEGFR-3 was moderately expressed in both MSS T4N3 and MSI T4N3 gastric cancers, while sparsely expressed in MSI T4N0 gastric cancers (Figure 2B). The breakdown of VEGF-C and VEGFR-3 levels assessed by IHC across 4 groups, namely, MSI T4N0, MSI T4N3, MSS T4N0, and MSS T4N3, is shown in Table S1.

### Subsets of T cells within microenvironment of gastric cancers

**Figure 3.**
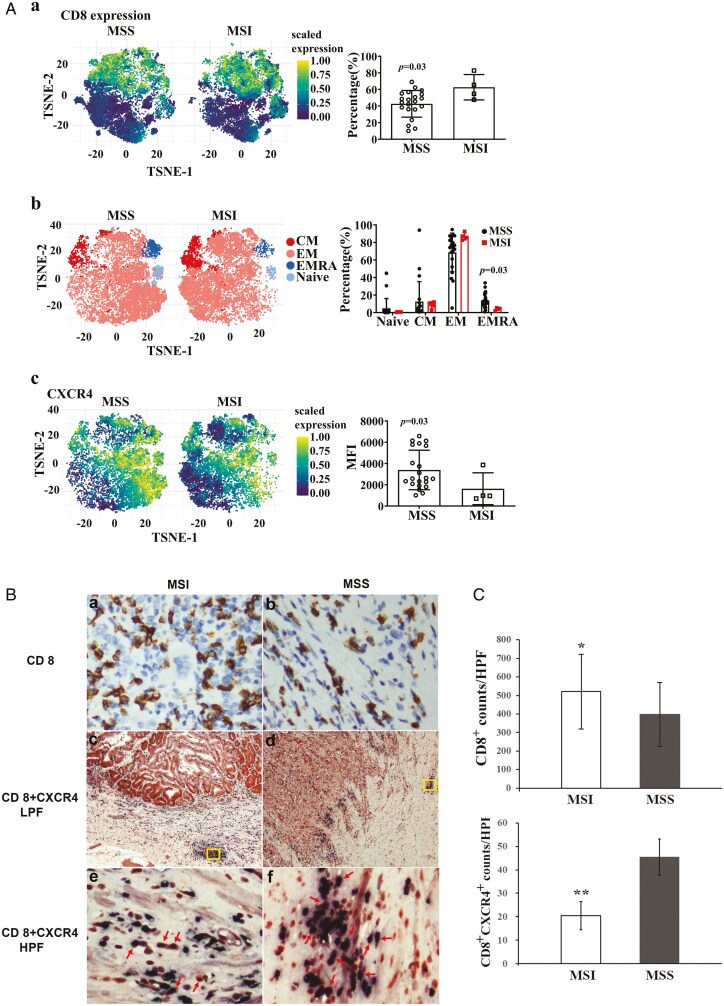
A. Subsets of CD 8+ T cells sorted by high-parameter flow cytometry among stage III gastric cancers with MSS (*n* = 22) vs MSI (*n* = 4): percentages of CD8+ (A); percentages of naïve, CM, EM, and EMRA (B); and expression of CD8+ CXCR4+ (C). B. Representative expression of CD8+ (a, b, upper panel) and CD8+ CXCR4+ (middle and lower panels) detected by immunohistochemistry among stage III gastric cancers with MSI (*n* = 10) vs MSS (*n* = 10). Using double stain technique, CD8+ appeared nuclear staining and CXCR4 appeared cytoplasmic staining. The features within rimmed boxes (c, d, middle panel) were amplified by 100-fold and shown in the lower panel (e, f), where arrows signaled CD8+ CXCR4+ T cells, which accessed to and aggregated around lympho-venules. LPF and HPF indicated low- and high-power field, respectively. C. Semi-quantification of CD8+ T cells and CD8+ CXCR4+ T cells detected by immunohistochemistry among stage III gastric cancers with MSI (*n* = 10) vs MSS (*n* = 10). *, ** Indicated *P* < .05 and .01, respectively.

After gating the T cells and performing dimension reduction using the t-SNE algorithm, the percentage of CD8+ T cells expression in the MSI group was increased as compared to MSS group (*P* = .03). Subsequently, we performed clustering analysis on the CD8+ T cells and characterized them into four distinct clusters based on specific markers, namely, naïve (CCR7+ CD45RA+), central memory (CM, CCR7+ CD45RA−), effector memory (EM, CD45RA− CCR7−), and effector cells expressing CD45RA (EMRA, CD45RA+ CCR7−). Through this analysis, we observed an increase in the EMRA population within the MSS group as compared to MSI group (*P* = .03). For a deeper understanding of CD8+ T cells egress from the TME, we investigated the differential expression of CXCR4 between the MSI and MSS groups, revealing a higher expression of CXCR4 in the MSS cases (*P* = .03; Figure 3A). IHC was employed to reaffirm the findings measured by the flow cytometry. MSI gastric cancers showed an increased total number of CD8+ T cells, albeit with a lower proportion of CD8+ CXCR4+ T cells, compared to MSS counterparts (Figure 3B). Semiquantitative analysis of CD8+ and CD8+ CXCR4+, respectively, among MSI and MSS gastric cancers are shown in Figure 3C.

## Discussion

Based on one of the largest single-institute surgical cohorts globally, we demonstrated that MSI gastric cancers were characterized by earlier N-stage, lower LN ratio, and earlier pathological stage at diagnosis compared to the MSS cohort, as thus reflected by superior survival at stage III of the former. Multivariate analysis showed that MSI status stood as an independent prognostic factor (HR, 1.8) following LN ratio (HR, 7.7-fold for the highest tier) and AJCC staging (HR, 2.5 for the highest tier). The LN ratio concept was originally introduced to address the drawback of insufficient lymph node dissection during gastrectomy and to prevent “stage migration.” However, the LN ratio has proven to be an independent prognosticator of gastric cancer, even surpassing the N-staging per se once the number of retrieved lymph nodes reaches an optimal level.^19^ In the present study, discrepancies in LN ratios between MSI and MSS gastric cancers increased with increasing T-staging; while the LN yields of both cohorts did not differ, indicating that MSI gastric cancers exhibited a higher negative lymph node count (NLNC). It has been reported that patients with a high NLNC and/or low LN ratio have a more favorable survival outcome.^20,21^ NLNC reflects the host’s immune response to the tumor, dependent on both the desmoplastic reaction and tumor immunogenicity. It is conceivable that a higher mutation load carried by MSI gastric cancers encodes abundant neo-antigens, which ignite and intensify immune reactions and the recruitment of TILs.^22–24^

The phenomenon of enriched NLNC carried by MSI gastric cancers had significant implications for clinical practice. MD CT remains the gold standard to determine the cN of gastric cancer and guide the management algorithm. Lymph nodes are considered metastatic in gastric cancer if the longest diameter is >1.0 cm or if the size is between 0.7 and 1.0 cm with hyper- enhancement, a round shape, central necrosis, or peri-nodal infiltration.^17^ Abiding by these criteria, cN among half of MSS gastric cancers was underestimated by our then-radiologists,^17^ whereas cN of the majority of MSI gastric cancers was otherwise overestimated. Most strikingly, up to one third of pT4 MSI gastric cancers turned out to be pN0, previously tentatively allocated as cN3 preoperatively and eligible for neoadjuvant chemotherapy using a fluorouracil-based regimen, which might not be beneficial.^5^

Central to our investigation is the role of VEGF-C, a pivotal regulator of lymphangiogenesis and lymphatic metastasis, through its interaction with VEGFR-3.^13^ Our data demonstrated differential expression patterns of VEGF-C and VEGFR-3 mRNA in MSI vs MSS gastric cancers, with lower expression levels observed in MSI ones. IHC analysis further revealed a gradient of VEGF-C expression across tumor stages, with higher levels in MSS T4N3 tumors compared to MSI counterparts and the disruption of VEGF-C -VEGFR-3 signaling among MSI T4N0. In addition to providing a venue for tumor cell spreading, overexpression of VEGF-C pushes lymphatic endothelial cells to take up and cross-present tumor antigens leading to dysfunctional activation of CD8+ T cells and rendering the TME immunosuppressive.^25,26^

CD8+ T cells infiltrating tumors are key to improving survival outcomes and response to immune checkpoint blockade.^27–29^ Given that the survival outcome of patients with MSI at stage III was far more favorable compared to their MSS counterparts, there must be causative factors beyond the T-N-M system, which we suggest are attributed to increased CD8+ T-cell infiltration within the TME. Dynamics of CD8+ T cells within the TME are controlled by intricate mechanisms of recruitment, retention, and exit via lymphatic vessels.^30^ T-cell recruitment is heavily dependent on tumor-specific antigens, whereas tumor-associated lymphatic vessels sequester CD8+ T cells, increasing the probability of exit in a CXCL12-CXCR4-dependent manner, promoting CD8+ T-cell egress out of the TME.^31–33^ Our data demonstrate an increased overall CD8+ T-cell presence in the MSI cohort compared to the MSS counterpart, as consistently shown by flow cytometry and IHC analyses. This heightened CD8+ T-cell presence in MSI gastric cancers underscores the robust anti-tumor immune response by the host toward these inherently neoantigen-enriched MSI tumors. By breaking down subsets of T cells, we observed an increase in the effector memory RA (EMRA) population within the MSS group compared to the MSI group, indicative of a more differentiated and potentially exhausted T-cell phenotype in MSS tumors.^34^ This suggests that the immune response in MSS tumors may be characterized by a shift toward a less cytotoxic and more regulatory T-cell profile, potentially contributing to immune evasion.^35^ Higher expression of CXCR4, a chemokine receptor associated with T-cell egress within the TME, was observed in MSS tumors, as measured by flow cytometry. Evidently, the differential distribution of CD8+ CXCR4+ T cells, with a more pronounced presence in MSS tumors and accessing peri-lymphovascular spaces as demonstrated by IHC, suggested a potential mechanism of immune evasion in MSS gastric cancers, where VEGF-C signaling might also play a role, as addressed above.

Clinically, although MSI gastric cancers generally have a better prognosis than their MSS counterparts, they exhibit a low pCR (pathological complete response) rate of only 3%-11% when treated with 5FU-based and platinum adjuvant regimens. In comparison, recent findings from the phase II NEONIPIGA trial demonstrated that a neoadjuvant combination of nivolumab and ipilimumab followed by adjuvant nivolumab is feasible in patients with locally advanced MSI gastric cancer, offering an acceptable toxicity (19%, grade 3/4 adverse events) and high pCR rate (58%) following curative surgery.^36^ This promising result raises an important question: How can we more accurately interpret the cN-staging of MSI gastric cancers to appropriately allocate patients to either upfront surgery or a neoadjuvant combined ICIs regimen?

While our study provides valuable insights, limitations such as the retrospective design and the absence of gain- and loss-of-function experiments warrant further investigation. Prospective studies with larger cohorts and mechanistic experiments are needed to validate our findings and elucidate the complex interactions between VEGF-C signaling and CD8+ T-cell dynamics. Another limitation is that our evaluation of peri-gastric lymphadenopathy in gastric cancers was based on MD CT, whereas endoscopic ultrasonography (EUS) was used for early gastric cancer cases potentially eligible for endoscopic resection.

In conclusion, our clinical observations highlighting overestimated cN by MD CT, decreased LN ratio, and increased NLNC in MSI gastric cancers, awarded by a better survival, fully depict the distinct characteristics compared to MSS counterparts. Mechanically, our investigation into VEGF-C signaling and CD8+ T-cell dynamics provides clues on how MSI gastric cancers take advantage and refrain from rapid disease deterioration and immune evasion, offering potential therapeutic targets aimed at modulating lymphatic metastasis and enhancing anti-tumor immune responses.

## Supplementary Material

oyae288_suppl_Supplementary_Figure

oyae288_suppl_Supplementary_Table

## Data Availability

The data presented here is available by contacting professor Ta-Sen Yeh, tsy471027@adm.cgmh.org.tw.
