# Supplementary figures and images for: Overestimation of clinical N-staging in microsatellite instable gastric cancers is associated with VEGF-C signaling and CD8+ T-cell dynamics
Source: Oncologist. 2024 Nov 18;30(7):oyae288. doi: 10.1093/oncolo/oyae288 (PMC12311297; doi:10.1093/oncolo/oyae288)

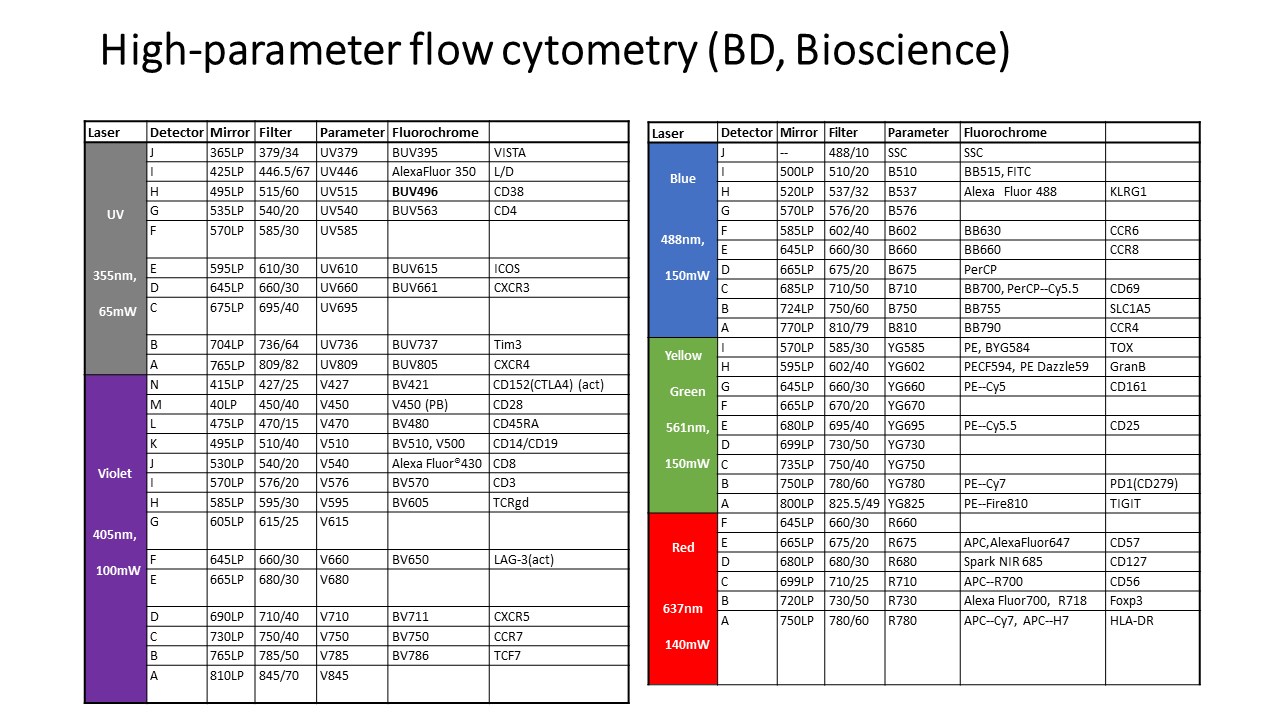

Supplement: oyae288_suppl_Supplementary_Figure [file oyae288_suppl_supplementary_figure.jpeg]
