# Supplementary material for: Overestimation of clinical N-staging in microsatellite instable gastric cancers is associated with VEGF-C signaling and CD8+ T-cell dynamics
Source: Oncologist. 2024 Nov 18;30(7):oyae288. doi: 10.1093/oncolo/oyae288 (PMC12311297; doi:10.1093/oncolo/oyae288)
Supplement: oyae288_suppl_Supplementary_Table [file oyae288_suppl_supplementary_table.docx]

| **Supplementary Table1.** Immunohistochemical expression of VEGF-C and VEGFR-3 among gastric cancers stratified by microsatellite status | | | | | |
| --- | --- | --- | --- | --- | --- |
| Parameters | MSI | |  | MSS | |
|  | T4N0  (n=10) | T4N3  (n=10) |  | T4N0  (n=10) | T4N3  (n=10) |
| VEGF-C | 4 | 6‡ |  | 8* | 10† |
| VEGFR-3 | 2 | 5‡ |  | 3 | 8†# |
| MSI and MSS indicated microsatellite instable and stable, respectively.  Immunopositivity of VEGF-C and VEGFR-3 was defined in Patient and Method section. | | | | | |

*, p<0.05 versus MSI T4N0; †, p<0.05 versus MSI T4N3; ‡, p<0.05 versus MSI T4N0; #, p<0.05 versus MSS T4N0
